# Beta catenin and cytokine pathway dysregulation in patients with manifestations of the "PTEN hamartoma tumor syndrome"

**DOI:** 10.1186/1471-2350-13-28

**Published:** 2012-04-20

**Authors:** Martina Galatola, Lorella Paparo, Francesca Duraturo, Mimmo Turano, Giovanni Battista Rossi, Paola Izzo, Marina De Rosa

**Affiliations:** 1 Dipartimento di Biochimica e Biotecnologie Mediche and CEINGE Biotecnologie Avanzate, Università di Napoli Federico II, via S. Pansini 5, Naples 80131, Italy; 2 Dipartimento di Biologia Strutturale e Funzionale, Università di Napoli Federico II, Complesso Universitario Monte S. Angelo, Via Cinthia, Naples 80126, Italy; 3 Istituto Nazionale dei Tumori - Fondazione G. Pascale, via M. Semmola, Naples 80131, Italy

**Keywords:** PTEN hamartoma tumor syndrome, *PTEN*, β-catenin, TNFα receptors

## Abstract

**Background:**

The "PTEN hamartoma tumor syndrome" (PHTS) includes a group of syndromes caused by germline mutations within the tumor suppressor gene "phosphatase and tensin homolog deleted on chromosome ten" (*PTEN*), characterized by multiple polyps in the gastrointestinal tract and by a highly increased risk of developing malignant tumours in many tissues.

The current work clarifies the molecular basis of PHTS in three unrelated Italian patients, and sheds light on molecular pathway disregulation constitutively associated to *PTEN *alteration.

**Methods:**

We performed a combination of RT-PCR, PCR, sequencing of the amplified fragments, Real Time PCR and western blot techniques.

**Results:**

Our data provide the first evidence of β-catenin accumulation in blood cells of patients with hereditary cancer syndrome caused by germ-line *PTEN *alteration. In addition, for the first time we show, in all PHTS patients analysed, alterations in the expression of TNFα, its receptors and IL-10. Importantly, the isoform of TNFRI that lacks the DEATH domain (TNFRSF1β) was found to be overexpressed.

**Conclusion:**

In light of our findings, we suggest that the PTEN pathway disregulation could determine, in non-neoplastic cells of PHTS patients, cell survival and pro-inflammatory stimulation, mediated by the expression of molecules such as β-catenin, TNFα and TNFα receptors, which could predispose these patients to the development of multiple cancers.

## Background

PTEN hamartoma tumor syndrome is the term that has recently been used to describe Cowden syndrome (CS), Bannayan-Riley-Ruvalcaba syndrome (BRRS), Proteus syndrome (PS), and Proteus-like syndrome. These disorders are all caused by mutations in the *PTEN *gene and are all characterized by extraintestinal manifestations in addition to intestinal polyposis. PHTS is inherited in an autosomal dominant manner, and is likely to be underdiagnosed because of its phenotypic variability, its incomplete penetrance, and the fact that many of its component features are subtle and occur in the general population [1].

CS is a rare multiple hamartoma syndrome with a reported incidence of 1 in 200,000 individuals. This syndrome is characterized by macrocephaly, mucocutaneous lesions (such as facial trichilemmoma), acral keratosis, and glycogenic acanthosis of the esophagus and papillomatous papules. It is also associated with thyroid, breast, and endometrial manifestations, including cancer in all of these areas. Renal cancer has also been associated with CS; the risk of developing gastrointestinal carcinoma in CS is of about 13% [2]. The BRRS is a congenital disorder characterized by macrocephaly, intestinal hamartomatous polyposis, lipomas, and pigmentated macules of the glans penis. It is recommended that individuals with BRRS should be considered at risk for malignancy, as with CS. PS is a complex, highly variable disorder involving congenital malformations and hamartomatous overgrowth of multiple tissues.

The diagnosis of PHTS is made only when a *PTEN *mutation is identified. Approximately 85% of individuals who meet the diagnostic criteria for CS and 65% of individuals with a clinical diagnosis of BRRS have a detectable *PTEN *gene mutation. Since the most serious consequences of PHTS relate to the increased risk of breast, thyroid, endometrial, and renal cancers, the most important aspect of managing individuals with a PTEN mutation is increased cancer surveillance [3,4].

*PTEN *is a 9-exon tumour suppressor gene that encodes for a 403 amino acid protein. It acts as a lipid phosphatase to negatively regulate the PI3K/AKT/mTOR Pathway [5,6]. Recently, nuclear compartmentalization of PTEN has been found as a key component of its tumor-suppressive activity [7]. Close to 100 different germ-line mutations of *PTEN *have been reported to date encompassing point, nonsense, frame shift, splice site, missense, and deletion/insertion mutations. Most mutations occur in exon 5, but mutations in all other exons, except the first, have also been described. Around 10% of *PTEN *mutations occur in the promoter region, and the role of epigenetic regulation is not well understood.

Somatic *PTEN *mutations have been identified in a large number of sporadic tumours such as glioblastomas, prostate cancer, melanomas, thyroid and endometrial tumours [3].

Evidence exists indicating that *PTEN *is a functionally haploinsufficient tumour suppressor gene [8]. Indeed, in human cancers, monoallelic mutation of *PTEN *without loss or mutation of the second allele is prevalent at an early stage, whereas complete loss is observed at low frequencies with the exception of advanced cancers [9,10]. Recent studies describe the functional relationship between mRNAs produced by the *PTEN *tumour suppressor gene and its pseudogene *PTENP1*, as well as the critical consequences of this interaction. These studies show that *PTENP1 *is biologically active as it can regulates cellular levels of *PTEN *and plays a growth-suppressive role [8].

In the current work, we present data obtained by studying three PHTS patients with a clinical diagnosis of CS (PHTS2 and PHTS3) and BRRS (PHTS1), respectively. The aim was to understand the pathogenetic mechanisms of the *PTEN *tumour suppressor gene alteration, and to clarify the molecular changes downstream of a *PTEN *alteration and constitutively associated to it.

## Methods

### Patients

A total of 3 unrelated patients, exhibiting hamartomatous polyposis, were referred by gastroenterologists to the laboratory, for genetic analysis. The histological aspect of the polyps was unambiguous in all cases and it is tipic of PHTS polyps. Differential diagnosis with the juvenile polyposis syndrome (JPS) was made for PHTS2 and PHTS3 patients because none of the patients showed polyps histologically characteristic of the JPS. In fact, in both patients polyps were hamartomas with glandular structures, muscle fibers and also ganglioneuromatosi elements, while Juvenile polyps show a normal epithelium with a dense stroma, an inflammatory infiltrate, and a smooth surface with dilated, mucus-filled cystic glands in the lamina propria. Muscle fibers and the proliferative characteristics of adenomas are typically not seen in juvenile polyps [11]. In addition, the PHTS2 patient showed extensive glycogenic acanthosis, that is a characteristic feature of Cowden syndrome [12], whereas, the PHTS3 patient showed horseshoe kidney. It was the combination of these elements that can be found in Cowden syndrome to suggest the diagnosis. Clinical phenotype, family history and molecular characterization of each patient are reported in Table 1.

**Table 1:** Clinical phenotype and molecular characterization of Italian PHTS patients studied

Patient	S/F	Age of diagnosis	Mutation	Clinical manifestations	Affected relatives
PHTS1	F	53 yrs	c.406 T→C p.C136R	BRRS: macrocephaly, brain asymmetry, arteriovenous malformations, glycogenic acanthosis, hamartomatous gastric polyps, colon cancer, penis macules, keratosis of the hands and feet	Father and one brother died of colon cancer; they both showed penis macules.

PHTS2	S	45 yrs	↓ mRNA	CS: hamartomatous gastric Cowden polyps, colon cancer, glycogenic acanthosis, keratosis of the hands and feet, a few freckles on penis	___

PHTS3	S	54 yrs	↓ mRNA	CS: hamartomatous colonic Cowden polyps, colon cancer, horseshoe kidney.	___

PHTS1, PHTS2 and PHTS3: patients affected by PHTS syndrome; F: familial cases; S: sporadic cases

Blood samples from healthy subjects were collected from the same hospital as the PHTS patients.

Samples from all families that participated in the study were collected after being granted authorization from the "Comitato etico per le attività Biomediche - Carlo Romano" of the University of Naples Federico II, with protocol number 120/10. Such authorization is given only once the study has received ethical approval, and participants' informed and written consent has been obtained.

### Molecular analysis of the PTEN messenger

#### RT-PCR of PTEN full length coding region in PHTS patients

Cells from from 3 ml of peripheral blood of these three PHTS patients were pelleted, total RNA was extracted using Trizol reagent (invitrogen, Life Technologies, Ca, USA), and cDNA was synthesized using 1 μg of total RNA, 500 ng of random hexamers and 1 μl Superscript III reverse transcriptase (INVITROGEN), in the presence of 4 μl 5X RT buffer, 1 μl DTT (0.1 M) and 1 mM dNTPs. The reaction was run for 50 mins at 42°C in a 20 μl reaction volume, heated to 70°C for 15 mins and quick chilled on ice. Next, 1 μl of the cDNA was amplified by PCR using the following couple of oligonucleotides:

PTEN-5'UTR-1FP: TTCCATCCTGCAGAAGAAGC [NM_000314.4]; start: +950;

PTEN-3'UTR-1RP: TCTGAGCATTCCCTCCATTC [NM_000314.4]; start: + 2765.

The amplification reaction produced a fragment of 1815 bp of molecular weight. The PCR products were analysed on a 1% agarose gel in a trisacetic acid (TAE)-EDTA standard buffer, and visualized by ethidium bromide staining.

#### Sequence analysis of PTEN messenger

Sequence analysis of the PTEN full length coding region was performed on amplified fragments from the cDNA of all three patients, using the following primer pairs:

PTEN-5'UTR2-FP: GCAGCTTCTGCCATCTCTCT; [NM_000314.4]; start: +980;

PTEN-7cRP: TCACCACACACAGGTAACGG; [NM_000314.4]; start: +1786;

PTEN 5cFP: TTGAAGACCATAACCCACCAC; [NM_000314.4]; start: +1300;

PTEN-8cRP: CCTTGTCATTATCTGCACGC; [NM_000314.4]; start: +1971;

PTEN-7cFP: CGACGGGAAGACAAGTTCAT; [NM_000314.4]; start: +1728;

PTEN-3'UTR2-RP: TAAAACGGGAAAGTGCCATC; [NM_000314.4]; start: +2530.

The analysis was performed in a 3100 Genetic Analyzer (Applied Biosystems, Foster City, CA, USA). For nucleotide numbering, the first A of the initiator ATG codon is nucleotide +1 of the *PTEN *mRNA sequence [GenBank Accession number NM_000314.4]. All oligonucleotides were obtained with primer-BLAST Software http://www.ncbi.nlm.nih.gov/tools/primer-blast/.

#### Real Time RT-PCR quantification analysis

Real Time PCR quantification analysis was performed for the *PTEN *messenger. The relative expression was calculated with the comparative Ct method. Patient-numbering corresponds to that adopted in Table 1. Three ml of peripheral blood cells from PHTS patients and healthy subjects were pelleted after erythrocytes lysis and resuspended in a Trizol reagent. In order to better normalize the healthy values, we used four blood mixes as controls, each containing five samples collected from healthy subjects, for a total of twenty controls (data not shown). In order to allow accurate evaluation of interindividual variability in expression, we also separately analysed 20 samples, collected from healty subjects. PHTS1, PHTS2 and PHTS3 are the three patients affected by PHTS syndrome, as reported in Table 1.

The *PTEN *mRNA quantification was carried out by amplifying fragments spanning the junctions between exons 5-6, compared to the glucuronidase transcript fragment, using the following oligonucleotides:

PTEN-5c2FP: ATGGGGAAGTAAGGACCAGAG; [NM_000314.4]; start: +1495;

PTEN-6cRP: TCTTGTGAAACAACAGTGCCA; [NM_000314.4]; start: +1623.

The quantitative Real Time assays were performed using the iCycler iQ Real Time Detection System (BIO-RAD). Amplification was carried out within a total volume of 15 μl containing the SYBR Green PCR Master Mix 1X (BIO-RAD), using 20 ng of cDNA. The Real Time PCR reaction was optimized according to the manufacturer's instructions but scaled down to 15 μl per reaction. The PCR conditions were standard and all reagents were contained in the standard iQ™ SYBR Green Supermix (BIORAD). The reaction protocol consisted in an initial predenaturation step at 95°C for 3 mins followed by 40 two-step cycles at 95°C for 15 s and 60°C for 1 min.

At the end of the PCR, the temperature was increased from 55 to 95°C at a rate of 3°C/min, and the fluorescence was measured every 10 s to construct the melting curve.

A nontemplate control was run for each assay, and all determinations were performed in triplicate to ensure reproducibility. Synthesis of the expected PCR product was confirmed by melting curve analysis. Oligonucleotides yielding 100-150 bp-long PCR products at an annealing temperature of 60°C were obtained with primer-BLAST Software http://www.ncbi.nlm.nih.gov/tools/primer-blast/.

### Molecolar analysis of PTEN gene and PTENP1 pseudogene

#### Genomic PCR and sequencing

Cells from 3 ml of peripheral blood of PHTS patients and healty controls were pelleted and genomic DNA was extracted using Nucleon BACC2 Kit (Amersham; Biosciences)

Genomic PCR and sequencing of exon 5 was performed for PHTS1 patient, using oligonucleotides complementary to intronic neighboring boundary regions of the exon

(PTENg5FP: TGTTAAGTTTGTATGCAACATTTCT; [NC_ 000010.10] start: 89692673;

PTENg5RP: AACCCAAAATCTGTTTTCCA; [NC_ 000010.10] start: 89693081), in order to distinguish the gene sequence from the pseudogene sequence. The GenBank Accession number of *PTEN *genomic sequence is: NC_000010.10.

Mutational analysis of PTEN promoter region, from bp -1398 to bp +1, was performed by PCR and sequencing. This region was amplified into two overlapping fragments of 663 and 789 bp in molecular weight, respectively, using the following primer pairs:

PTENp-FP: GTTTCTCGCCTCCTCTTCGT; [NC_ 000010.10] start: 89623575;

PTENp-RP: ATGGCTGTCATGTCTGGGA; [NC_ 000010.10] start: 89624237;

PTENp2-FP CTACACTGAGCAGCGTGGTC: [NC_ 000010.10] start: 89622825;

PTENp2-RP GGCTGCACGGTTAGAAAAGA; [NC_ 000010.10] start: 89623613.

#### Gene copy number quantification of PTEN gene and PTENP1 pseudogene

For the genomic quantification of *PTEN *gene and *PTENP1 *pseudogene, specific amplified fragments were compared to a fragment of the exon 15 *MUTYH *gene. For *PTEN *specific quantification, two short fragments, one inside intron 9 and the other at the boundaries of exon 5 and IVS5, were amplified, using the following primer pairs:

PTEN-5c2FP: ATGGGGAAGTAAGGACCAGAG; [NM_000314.4]; start: 1495;

PTENg5RP: AACCCAAAATCTGTTTTCCA; [NC_ 000010.10] start: 89693081;

PTEN-IVS9-FP:AGGCCTCTTAAAGATCATGTTTGT; [NC_ 000010.10] start: 89724893;

PTEN-IVS9-RP:AGAAAATTTAAAATGAAAACCCACA; [NC_ 000010.10] start: 89725035.

These oligonucleotides don't recognize the *PTENP1 *sequence, whereas for specific amplification of the 3'-UTR of *PTENP1*, the primers described by Poliseno et al. [8] were used. H1, H2 and H3 are three healthy subjects supposedly not deleted for the target genes.

### Molecular alterations of PI3K/akt and WNT pathways associated to PHTS syndrome

#### Western blot assay of β-catenin protein

Total proteins were extracted from 3 ml of peripheral blood cells (about 5-7 × 10^3^/mL cells) using Trizol reagent (invitrogen, Life Technologies, Ca, USA) following the manufacturer's instructions; concentrations were determined by using a protein assay kit adopting bovine serum albumin standards, according to the manufacturer's instructions (Bio-Rad Laboratories, Hercules, CA, USA). A total of 50 μg protein was separated by SDS-polyacrylamide gel electrophoresis and blots were prepared on a nitrocellulose membrane Amersham Hybond-ECL (Amersham, GE healthcare, USA). The primary antibodies against β-catenin (amino-terminal and phospho beta catenin-S552 antigen) were from Cell Signaling technology (Beverly, MA, USA). The antibody against actin was from Santa Cruz (Santa Cruz, CA, USA). The membrane was probed with a secondary antibody against peroxidase-conjugated rabbit and goat immunoglobulin G, and immunoreactive bands were detected using the enhanced chemiluminescence HRP Substrate Immobilon Western, Millipore (Millipore Corporation, Billerica, USA). H_1-5_, H_6-10_, H_11-15_, H_15-20 _were mixes of healthy subjects, performed as described before. PHTS1, PHTS2 and PHTS3 were the patients affected by PHTS syndrome, as reported in Table 1.

#### Real Time PCR quantification analysis of COX2, CCND1, cMYC, and APC messengers

Relative expression was calculated with the comparative Ct method and normalised against the Ct of Glucuronidase (*GUS*) messenger. The quantitative RNA Real Time assays were performed as described before. H_1-5_, H_6-10_, H_11-15_, H_15-20 _were mixes of healthy subjects. H_m _was the mean value between all healthy samples used as calibrator to measure the relative expression. PHTS1, PHTS2 and PHTS3 were the three patients affected by PHTS syndrome, as reported in Table 1.

### Cytokine disregulation on peripheral blood cells of PHTS patients

#### Real Time PCR quantification analysis of IL10 and TNFa mRNA

Messenger quantification was performed for *IL10 *and T*NFa *and normalised against the Ct of Glucuronidase (*GUS*) messenger. The assay was performed as described before.

#### Western blot assay of TNFRI and TNFRII proteins

Primary antibodies against TNFRI and TNFRII were from R&D System (R&D System, Minneapolis, USA). The antibody against actin was from Santa Cruz (Santa Cruz, CA, USA). Secondary antibodies were against peroxidase-conjugated goat and mouse immunoglobulin G. The assay was performed as described before.

H_1-5_, H_6-10_, H_11-15_, H_15-20 _were mixes of healthy subjects, performed as before. PHTS1, PHTS2 and PHTS3 were the three patients affected by PHTS syndrome, as reported in Table 1.

### RT-PCR and sequencing of TNFRIAβ

Messenger expression of the beta isoform of TNFRIA was analysed by RT-PCR and sequencing. One μl of the cDNA syntetized from RNA of each PHTS patient was amplified using the following couple of oligonucleotides:

TNFRI-5cFP: AAACAGAACACCGTGTGCAC;

TNFRI-6BcRP: CTTGAATCTGGGAGGCAGAG.

Sequence analysis was performed using the same primer pairs, as previously described. The GeneBank accession number of TNFR1Aβ is: EU927389.1.

### Statistical analysis

All statistical analyses were computed using Prism software. The unpaired two-tailed *t *test with confident intervals of 99% was calculated. A value of *p <*0.005 was considered statistically significant.

## Results

### Molecular analysis of the *PTEN *gene at DNA and RNA level

We performed mutational analysis of the PTEN gene, setting up a combination of RT-PCR reaction of the whole cDNA, PCR of genomic region, including the promoter region from bp -1398 to bp +1, sequencing of the amplified fragments and Real Time PCR.

**Figure 1:**
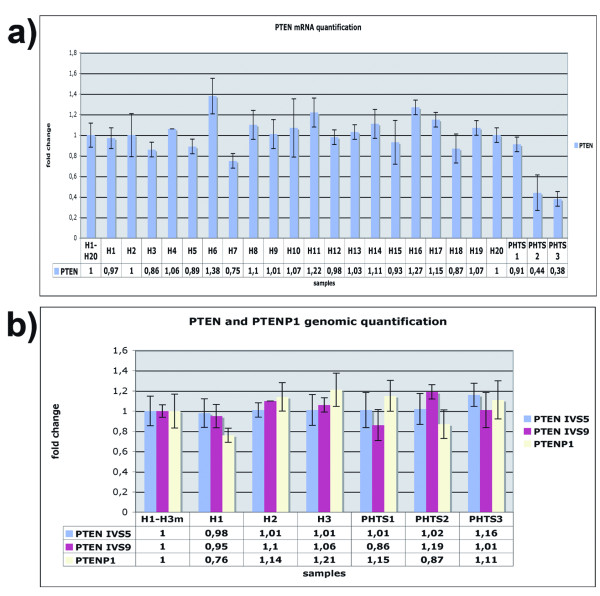
**Alterations of PTEN gene in PHTS patients**. *a) Real Time PCR quantification analysis of PTEN mRNA*. Real Time RT-PCR quantification analysis was performed for the *PTEN *messenger. H_1-20_: mean value between all healthy samples used as calibrator to measure the relative expression; H_1_, H_2_, ... H_20_: healty subject supposedly not deleted for the target genes. The graph shows quantification of the *PTEN *mRNA of the PHTS patients and twenty healty subjects. *b) Copy number quantification of PTEN gene and PTENP1 pseudogene*. Real Time PCR quantification analysis was performed for *PTEN *and *PTENP1 *genomic sequences. IVS9: amplified fragment inside intron 9 of the PTEN gene; IVS5: amplified fragment at the boundaries of exon 5 and IVS5 of the PTEN gene; PHTS1, PHTS2 and PHTS3: patients affected by PHTS syndrome, as reported in Table 1. H1, H2 and H3: three healthy subjects supposedly not deleted for the target genes.

As shown in Table 1 patient PHTS1, affected by BRRS, had a missense mutation named c.406T→C in exon 5 of *PTEN *gene. This mutation, already described in the literature, determines the aminoacidic change of a cysteine residue at position 136 into an arginine [13,14]. The other two patients, PHTS2 and PHTS3 (both affected by CS), showed a significant decrease in the *PTEN *mRNA expression when analysed by Real Time quantitative RT-PCR (Figure 1a) (unpaired two-tailed *t *test, confident intervals 99% p: 0,0002); this statistic result was the same when calculated with the twenty healty controls separately and with the four mixes of healty subject (data not shown). None of these patients was a carrier of a PTEN point mutation of the promoter or coding gene region, nor did they show intragenic or whole gene deletion when analysed by Real Time genomic PCR (Figure 1b). However, promoter iper-methylation cannot be excluded, and additional mechanisms could be responsible for the PTEN down-expression in these two patients. For example, point mutations in gene regions not investigated, such as intronic regions and regions at the 5' and 3' end of the gene (not included in our analysis), or mutations in other genes that could regulate PTEN expression, could be present in these patients. Using the same technique, we also performed a genomic quantification of the *PTENP1 *pseudogene, in light of its involvment in the *PTEN *messenger regulation, the quantity of which still resulted normal in all patients (Figure 1b) (unpaired two-tailed *t *test, confident intervals 99%; p_PTEN_: 0,9161; p_PTENP1_: 0,7755).

### Molecular alterations of PI3K/akt and WNT pathways are observed in non-neoplastic cells of PHTS patients

To better clarify the patients' molecular changes associated to a germline *PTEN *alteration, we studied some genes related to PTEN/PI3K/AKT molecular pathway or WNT pathway, and typically involved in tumour development. Indeed, a relatively large number of studies indicate a cooperative effect of these two molecular pathways [15]. It is already known that β-catenin phosphorylation at Ser-552, promoted by PI3K-Akt signalling, is associated with its stabilization, nuclear accumulation and transcriptional activation. Furthermore, PI3K-Akt signalling activation results in the inactivation of GSK-3β and reduces N-terminal β-catenin phosphorylation, which is associated with its degradation [16,17].

**Figure 2:**
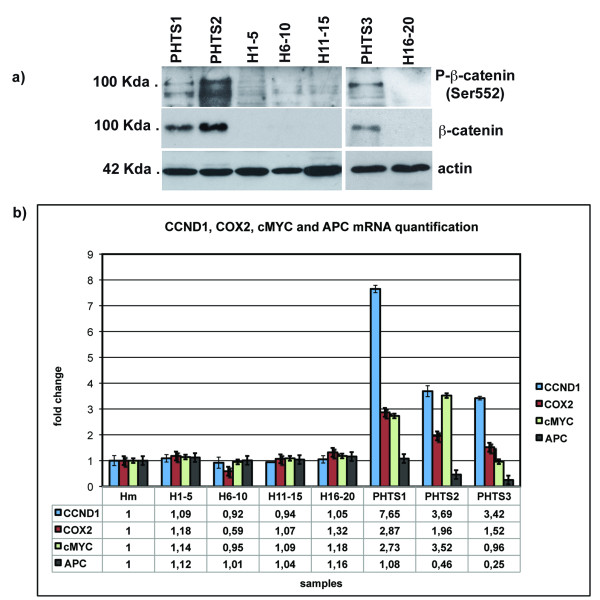
**Molecular alterations of PI3K/Akt and WNT pathways associated with PHTS syndrome**. *a) Western blot assay of β-catenin protein*. H_1-5_, H_6-10_, H_11-15_, H_15-20_: mixes of healthy subjects. PHTS1, PHTS2 and PHTS3: patients affected by PHTS syndrome, as reported in Table 1. *b) Real Time PCR quantification analysis of COX2, CCND1, cMYC, and APC messengers. H*_*1*-5_, H_6-10_, H_11-15_, H_15-20_: mixes of healthy subjects; H_m_: mean value between all healthy samples used as calibrator to measure the relative expression; PHTS1, PHTS2 and PHTS3: patients affected by PHTS syndrome, as reported in Table 1.

Thus, we performed a western blot analysis of β-catenin on protein extracted from non-neoplastic peripheral blood cells of PHTS patients and healthy subjects, to verify whether *PTEN *alteration causes β-catenin intracellular accumulation in these cells. As shown in Figure 2a, β-catenin is detectable by using N-terminal antibody and phospho-β-catenin (Ser552) only in PHTS patients; total β-catenin (detected by N-terminal antibody) is absent in all healthy controls analysed, while phospho-β-catenin (Ser552) is detected mainly in PHTS patients and gives a very faint signal in controls. The phospho-β-catenin (Ser552) accumulation favors a model whereby de-regulation of the PI3K/Akt signaling is responsabile for alteration of the β-catenin expression, while total β-catenin accumulation suggested a possible transcriptional activation of its target genes. In order to evaluate this hypothesis, we performed Real Time RT-PCR experiments for *c-MYC, cycline D1 *(*CCND1*) and *COX2 *messenger, classically under the control of b-catenin transcription factor. Following this, we also analysed the *APC *gene expression, as it is known to be the major regulator of the WNT pathway. As expected, quantification of *cMYC, CCND1*, and *COX2 *messengers, conducted on RNA extracted from peripheral blood cells of the three PHTS patients and healthy subjects, revealed an mRNA overexpression of all these genes in the patients when compared with the healthy subjects - with the exception of patient PHTS3, who expresses a normal level of *cMYC *RNA (Figure 2b) (unpaired two-tailed *t *test, confident intervals 99%: p_cMYC _= 0,0001; p_CCND1 _= 0,0001; p_COX2 _= 0,0001). As showed in Figure 2b, the *APC *transcript resulted down-expressed only in patients PHTS2 and PHTS3 (affected by CS and carrier of *PTEN *mRNA down-expression), again as compared with the healthy controls (unpaired two-tailed *t *test, confident intervals 99%: p_APC _= 0,0001). The relative espression values of each transcript analysed, normalised versus the glucuronidase (*GUS*) transcript, are reported in Figure 2b.

### Cytokine dysregulation is observed in non-neoplastic cells of PHTS patients

**Figure 3:**
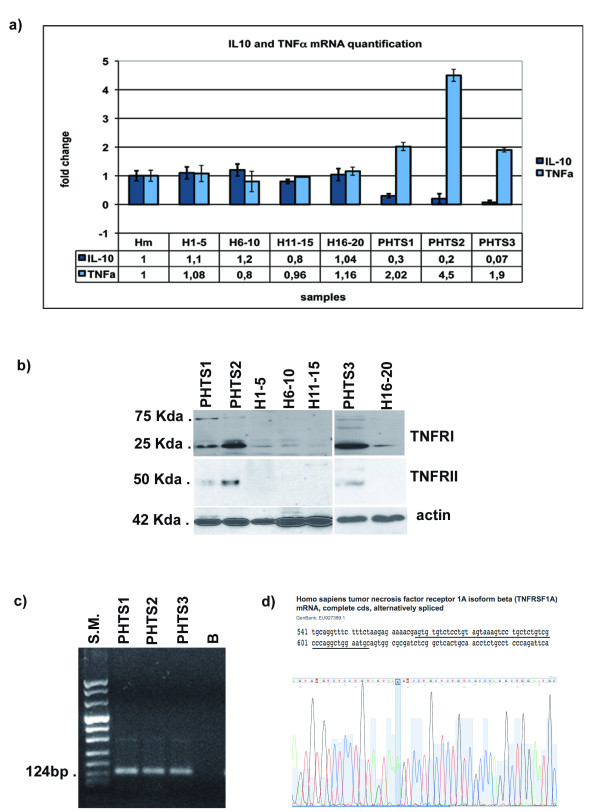
**Cytokine disregulation on peripheral blood cells of PHTS patients***a) Real Time PCR quantification analysis of IL10 and TNFα mRNA*. *b) Western blot assay of TNFR1 and TNFR2*. H_1-5_, H_6-10_, H_11-15_, H_15-20_: mixes of healthy subjects; H_m_: mean value between all healthy samples used as calibrator to measure the relative expression; PHTS1, PHTS2 and PHTS3: patients affected by PHTS syndrome, as reported in Table 1. *c) RT-PCR analysis of the TNFRSF1Aβ isoform*. S.M.: genomic molecular weight marker; B: amplified sample without cDNA template. *d) sequence analysis of the amplified fragments showed in *Figure 3d. The genomic sequence GenBank accession number is: EU927389. The underlined nucleotide are those showed in the electropherogram.

Finally, we performed Real Time mRNA quantification of the *IL10 *and *TNFa *messengers and western blot analysis of TNFARI and TNFARII. The relative quantification of the *IL-10 *and *TNFa *mRNA revealed statistically significant downregulation of *IL-10 *mRNA and upregulation of *TNFa *mRNA in PHTS patients when compared with healthy controls (Figure 3a) (unpaired two-tailed *t *test, confident intervals 99%: p_IL-10 _= 0,0001; p_TNF _= 0,0001).

As shown in Figure 3b, TNFARI and TNFRII proteins were detectable mainly in the patients, in our experimental conditions. Interestingly, the main western blot signal obtained by using anti-TNFRI antibody in all three patients highlights a protein of about 25 Kda that corresponds to the isoform β of the TNFR1A, described by Wang et al. [18]. This isoform, generated by an alternative splicing mechanism and designated as *TNFRSF1Aβ *[genomic sequence GenBank accession number: EU927389; protein sequence GenBank accession number: NP_001056], lacks the trans-membrane helix and the full cytoplasmic region including the DEATH domain compared to the full-length protein, while retains the signal peptide and the conserved binding domain, which corresponds to the TNFR (TNF receptor) domain [18]. We have confirmed, by RT-PCR and sequencing, that mRNA for this *TNFRIA *isoform was expressed in our samples (Figure c and d).

## Discussion

It is becoming increasingly evident that inflammation and cancer are intricately related. Many cancers arise from sites of infection, chronic irritation and inflammation [19-21]. Two of the key molecules mediating the inflammatory processes in tumour promotion are cytokines tumour necrosis factor-α (*TNF-a*), and *IL10 *[22-24]. Moreover, much data in the literature correlate *TNFa *expression with *PTEN *gene expression [25-29]. Usually, the TNFα induces apoptosis through TNFRI and functions related to cell survival through TNFRII. The TNFα binding to TNFRI activates apoptosis through two pathways, involving the adaptor proteins TNFRI-associated death domain (TRADD), and Fas associated death domain (FADD). By contrast, TNFRII signalling involves the mobilization and nuclear entry of the transcription factor nuclear factor-κB (NF-κB) [30,31]. However, receptor crosstalk and overlapping functions are described [31,32].

It is reasonable to hypothesize that the expression of the isoform b of the TNFRIA observed in the PHTS patients could represent a mechanism by which TNFRI, lacking its DEATH domain, could shift from an apoptotic signal towards cell survival stimulation, thus inducing tumour promotion. Additional experiments are necessary to clarify the role that TNFRIAβ could play in the TNFα signalling.

The relationship between PI3K activation and the TNFα pathway is controversial. Evidence exists that the PI3K-Akt pathway negatively regulates NF-kB and the expression of inflammatory genes [33,34], as well as that PI3K activation induces TNFα over-expression in different cell types [35,36]. This last finding are in agreement with our data. We cannot exclude that alterations of molecular pathways, such as the IL10 and TNF pathways, showed in non-neoplastc peripheral blood cells of PHTS patients are related to specific roles that PTEN plays in the nucleus, independent of the PI3K/Akt pathway [7].

These data represent the first evidence of β-catenin accumulation in non-neoplastic cells of PHTS patients, caused by germ-line *PTEN *alteration without a "second hit" of gene inactivation taking place. In light of these data, we suggest that β-catenin could represent a good candidate as a diagnostic marker for hereditary colorectal diseases that determine β-catenin accumulation. This is mainly noteworthy for PHTS syndrome, which is often underdiagnosed. An expansion of the current study, to be conducted on a larger population, has been planned. This should confirm the robustness of the data presented herein.

Overall, our data is in line with several previous studies. In human alveolar macrophages, bacterial lipopolysaccharide (LPS) exposure activates PI3-K/Akt, inactivates GSK-3β, and causes accumulation of β-catenin in the nucleus of any cell and expression of genes that require nuclear

β-catenin for their activation [37]. In human endometrial cancer cells with *PTEN *mutation, the PI3K/Akt survival pathway is involved in the regulation of COX-2 and PGE2 synthesis. The authors showed that Akt phosphorylation was high in mutated *PTEN *cells compared to wild-type *PTEN *cells, and that this phosphorylation status is associated with overexpression of *COX-2 *mRNA, its protein levels, and prostaglandin E2 (PGE2) production [38]. In colorectal cancer, *PTEN *knockdown results in nuclear β-catenin accumulation and in increased expression of downstream proteins *c-MYC *and *cycline D1 *[28]. Moreover, Lee et al. demonstrated that, during inflammatory bowel disease, PI3K/Akt signalling cooperates with Wnt to increase β-catenin signalling, determining the progression from chronic ulcerative colitis to colitis-associated cancer [15].

In this scenario, the *APC *transcriptional down-regulation we have observed in peripheral blood cells of two PHTS patients, suggests an intrinsic cooperative action of PI3K/Akt and Wnt signalling to increase β-catenin intacellular concentration. Taking the latter into account, and as previously described for other tumour suppressor genes [39,40], *PTEN *mRNA molecular screening investigations are necessary to obtain more precise molecular diagnoses, with a view to better characterizing PHTS patients and probably to explain the PHTS cases that remain without identified molecular alterations. It is interesting that the reduced APC levels are found in the two cases that do not have a PTEN mutation identified on DNA. We can not exclude that this could be due to alternative mechanisms (possibly mutations in other genes), that cause reduction in both APC and PTEN mRNA level. Infact, though unlikely, the presence of genomic rearrangements in PHTS2 and PHTS3, cannot be completely ruled out with the methods used.

## Conclusion

In conclusion, it is likely that *PTEN *germ-line alteration and β-catenin accumulation, observed in peripheral blood cells of PHTS patients, could determine a cell survival and pro-inflammatory stimulation mediated by the expression of molecules such as *COX2, CCND1 *and *TNFa *receptors. Increased levels of messengers cMYC and CCND1 are often observed in different tumour types, such as breast cancer, endometrial cancer, thyroid cancer and ovarian cancer [41-43]. Interestingly, these types of tumours correspond to the neoplasias most frequently associated to PHTS syndrome. It is likely that the overexpression of mRNA of cMYC and CCND1 in non neoplastic cells of PHTS patients puts them at hight risk of developing these types of tumours.

The data presented herein are in agreement with and reinforces the results of Alimonti et al. [10], who propose a new "continuum model" for cancer initiation and promotion for the *PTEN *tumour suppressor gene, resulting from the subtle reduction in a tumour suppressor gene without requiring an additional genetic hit at that locus. In their model, tumours initiated by a subtle downregulation of a tumour suppressor gene can progress in the absence of LOH of the wild type allele [10].

Knowledge of specific molecular pathways changes downstream of *PTEN *alteration could be helpful in optimizing molecular targeted therapy and preventative care, not only for PHTS patients, but mainly because *PTEN *mutations are involved in a wide range of human diseases and cancers.

## Abbreviations

PI3K: Phosphatidylinositol 3-kinase; AKT: Protein kinase B-alpha; mTOR: Mammalian target of rapamycin; WNT: wingless-type MMTV integration site family; cMYC: V-myc avian myelocytomatosis viral oncogene homolog; CCND1: Cycline D1; COX2: Cyclooxygenase 2; APC: Adenomatous polyposis coli; TNF-α: Tumor necrosis factor alpha; TNFRI: Tumor necrosis factor receptor superfamily member 1A; TNFRII: Tumor necrosis factor receptor superfamily member 1B; IL-10: Interleukin 10.

## Competing interests

The authors declare that they have no competing interests.

## Authors' contributions

MDR: designed and processed the study, interpreted the data and wrote the first draft of the manuscript; performed statistical analysis and study supervision; conceived and supervised the study. MG: contributed in Real Time PCR experiment. LP and FD: contributed in PCR and sequencing. MT: contributed on western Blot; GBR: was responsible for endoscopy and clinical diagnosis of the patients; PI: critically evaluated the study and manuscript revision; obtained funding. All authors read and approved the final manuscript.

## Pre-publication history

The pre-publication history for this paper can be accessed here:

http://www.biomedcentral.com/1471-2350/13/28/prepub
